# Variability of radiotherapy volume delineation: PSMA PET/MRI and MRI based clinical target volume and lymph node target volume for high-risk prostate cancer

**DOI:** 10.1186/s40644-022-00518-7

**Published:** 2023-01-04

**Authors:** Lin-Lin Liu, Lei-Lei Zhu, Zhen-Guo Lu, Jun-Die Sun, Jun Zhao, Hai-Feng Wang, Zuo-Lin Xiang

**Affiliations:** 1 Department of Radiation Oncology, Shanghai East Hospital, School of Medicine, Tongji University, Shanghai, 200120 China; 2 Department of Nuclear Medicine, Shanghai East Hospital, School of Medicine, Tongji University, Shanghai, 200120 China; 3 Department of Urology, Shanghai East Hospital, School of Medicine, Tongji University, Shanghai, 200120 China; 4 Department of Radiation Oncology, Shanghai East Hospital Ji’an hostipal, Ji’an City, Jiangxi Province 343000 China

**Keywords:** Prostate cancer, PET/MRI, PSMA, Radiotherapy, CTV, GTVn

## Abstract

**Purpose:**

A comparative retrospective study to assess the impact of PSMA Ligand PET/MRI ([68 Ga]-Ga-PSMA-11 and [18F]-F-PSMA-1007 PET/MRI) as a new method of target delineation compared to conventional imaging on whole-pelvis radiotherapy for high-risk prostate cancer (PCa).

**Patients and methods:**

Forty-nine patients with primary high-risk PCa completed the whole-pelvis radiotherapy plan based on PSMA PET/MRI and MRI. The primary endpoint compared the size and overlap of clinical target volume (CTV) and nodal gross tumour volume (GTVn) based on PSMA PET/MRI and MRI. The diagnostic performance of two methods for pelvic lymph node metastasis (PLNM) was evaluated.

**Results:**

In the radiotherapy planning for high-risk PCa patients, there was a significant correlation between MRI-CTV and PET/MRI-CTV (*P* = 0.005), as well as between MRI-GTVn and PET/MRI-GTVn (*P* < 0.001). There are non-significant differences in the CTV and GTVn based on MRI and PET/MRI images (*P* = 0.660, *P* = 0.650, respectively). The conformity index (CI), lesion coverage factor (LCF) and Dice similarity coefficient (DSC) of CTVs were 0.999, 0.953 and 0.954. The CI, LCF and DSC of GTVns were 0.927, 0.284, and 0.32. Based on pathological lymph node analysis of 463 lymph nodes from 37 patients, the sensitivity, specificity of PET/MRI in the diagnosis of PLNM were 77.78% and 99.76%, respectively, which were higher than those of MRI (*P* = 0.011). Eight high-risk PCa patients who finished PSMA PET/MRI changed their N or M stage.

**Conclusion:**

The CTV delineated based on PET/MRI and MRI differ little. The GTVn delineated based on PET/MRI encompasses metastatic pelvic lymph nodes more accurately than MRI and avoids covering pelvic lymph nodes without metastasis. We emphasize the utility of PET/MRI fusion images in GTVn delineation in whole pelvic radiotherapy for PCa. The use of PSMA PET/MRI aids in the realization of more individual and precise radiotherapy for PCa.

**Supplementary Information:**

The online version contains supplementary material available at 10.1186/s40644-022-00518-7.

## Introduction

Prostate cancer (PCa) is a common malignancy in men, accounting for 27% (233,000) of cancer incidence in the United States [1]. In recent years, the incidence of prostate cancer in China has significantly increased, seriously affecting men’s health; the most common pathological type of PCa is adenocarcinoma [2]. Lymphatic and haematogenous metastasis are the two common metastatic pathways of prostate cancer, and the metastasis of pelvic lymph nodes (PLN) is strongly associated with the prognosis of prostate cancer [3].

PCa cases are stratified into low/intermediate-risk and high-risk groups according to the definition of the European Association of Urology (EAU) based on the serum prostate specific antigen (PSA) level, Gleason score and clinical stage of prostate cancer patients. Among them, high-risk localized prostate cancer includes patients with prostate-specific antigen (PSA) > 20 ng/ml or Gleason score > 7 (Gleason Grade Group 4/5) or cT2c [4].

Radical prostatectomy, radiation therapy (with or without androgen deprivation therapy), androgen deprivation therapy, deferred treatment (active surveillance) or watchful waiting are the current treatment strategies for prostate cancer [5, 6]. Among them, radiotherapy is one of the main treatment methods for organ-confined and locally advanced prostate cancer, along with hormone therapy and surgery. And it has an irreplaceable role in improving the survival rate, prolonging the survival time and reducing complaints of patients with prostate cancer [7–9]. For high-risk prostate cancer (clinical T1-4N0-1M0), radical radiotherapy combined with endocrine therapy can achieve the same efficacy as surgery [10]. Schaeffer E confirmed that prophylactic whole-pelvis radiation improves disease-free survival and biochemical-failure-free survival compared with prostate radiotherapy alone for high-risk, locally advanced prostate cancer [11]. Whole-pelvis radiation is essential in high-risk prostate cancer treatment and is closely associated with actual survival and prognosis.

Accurate outlining of the target area is the cornerstone to ensure radiotherapy efficacy. Precise external radiotherapy improves in-field tumour control while reducing the incidence of toxic side effects. Currently, computed tomography (CT) and magnetic resonance imaging (MRI) are widely used for target volume delineation [12], and MRI has the advantage of better soft tissue resolution, multidirectional imaging techniques and no ionizing radiation compared to CT. However, CT or MRI can show the anatomical features of the tumour but provide little information regarding the tumour's biological behavior. The introduction of positron emission tomography (PET) tracers targeting prostate-specific membrane antigen (PSMA) has filled this gap. PSMA, as an important biomarker, is a type II transmembrane glycoprotein that is expressed at 100–1000-fold higher levels in prostate cancer cells than in normal cells [13]. Several studies have shown that PSMA-targeted PET is superior to conventional imaging (CT, MRI and 18F-FDG) in detecting metastases, and some articles have confirmed that PSMA PET/MRI is superior to multiparametric MRI in diagnosing prostate cancer [14–18]. PET/MRI combines the advantages of PET and MRI, providing anatomical images in a single imaging session, detailed functional and cellular metabolism and other molecular information of the lesion, while the anatomical structure of the lesion can be accurately displayed, providing more clinical information compared to PET/CT. However, there is a lack of evaluation of the value of PSMA PET/MRI in the clinical radiotherapy of prostate cancer.

The aim of this study was indeed to evaluate the difference in CTV and GTVn outlined by MRI and PET/MRI fusion images to investigate the value of PSMA PET/MRI in high-risk prostate cancer radiotherapy.

## Methods

### Data collection

**Fig. 1:**
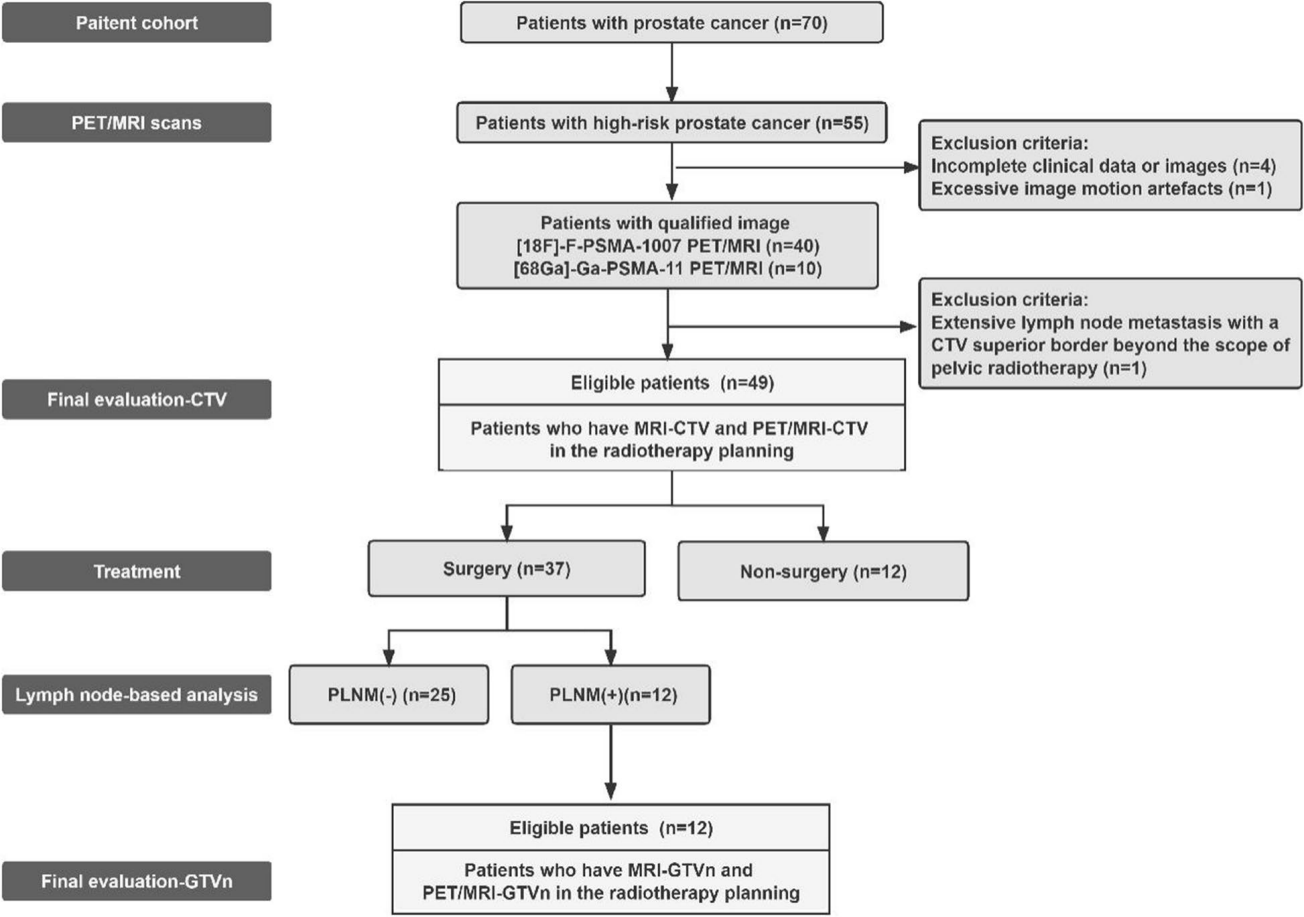
Patient enrollment process

From May 2020 to December 2021, we retrospectively enrolled 70 patients with definitive prostate cancer by pathological biopsy who presented to our hospital for PSMA PET/MRI. The patient enrollment process is summarized in Fig. 1. All patients had received no other treatment prior to this date, and no patients had contraindications to MR or PET imaging. Of the 70 patients, 55 cases were defined as having high-risk prostate cancer according to the European Association of Urology guidelines. 4 cases were excluded due to incomplete clinical data or images, 1 case was excluded due to excessive image motion artefacts and 1 case was excluded due to its prostate cancer metastasis exceeding the upper bound of the CTV. 49 patients were finally included in this study, 39 of whom underwent [18F]-F-PSMA-1007 PET/MRI imaging, and the other 10 underwent [68 Ga]-Ga-PSMA-11 imaging. The study was approved by our ethical review committee.

### PET/MRI Image Acquisition

Patients should be encouraged to drink sufficient amounts of water and to empty their bladders prior to and after the PET/MRI examination. 0.1 mCi/kg body weight of 18F-PSMA-1007 or 68 Ga-PSMA-11 was injected intravenously. Then, whole-body PET scans were performed, covering 5 bed positions with an acquisition time of 3 min per bed position (15 min at the prostate bed position). Hybrid PET/MRI images were acquired in 3D mode on a 3-Tesla PET/MR scanner (uPMR790 TOF, United Imaging, China). PET images were reconstructed by the ordered subset expectation maximization (OSEM) algorithm (including 2 iterations, 20 subsets, a 4 mm full-width half-maximum (FWHM) Gaussian filter, and a 150 × 150 image matrix). The device uses the tissue segmentation method for attenuation correction, acquiring images with a 2-point Dioxon sequence and dividing the images into air, lung, fat, and soft tissue for attenuation correction. Diagnostic MRI scans included T1-weighted high-resolution isotropic volume acquisition, T2-weighted (T2W) 3D volumetric fast spin-echo (FSE) imaging in the axial, sagittal, and coronal directions. T1 sequence parameters: repetition time (TR)/echo time (TE) = 5.04/2.24 ms, 4 mm slice thickness, 20% interslice gap, 350 mm × Ax FSE T2 sequence parameters: TR/TE = 3998/88.74 ms, 6 mm slice thickness, 20% interslice gap, 300 mm × 300 mm field of view, 320 × 320 matrix. Delayed pelvic PET/MRI scans were performed if clinically indicated. Image analysis was performed to confirm the MRI and PET/MRI images of each patient separately, which was performed by 2 radiologists, one working in radiology and the other in nuclear medicine, both board-certified and with more than 5 years of experience. The three radiation oncologists further performed target area outlining independently based on the above, including the patient's tumour target volume (GTV) and clinical target volume (CTV). None of the three physicians had any prior medical involvement, knowledge of patients’ medical history, or had previously seen any images of them. A medical report of the course of illness and written reports from the radiologist and nuclear medicine physician were also provided, and the imaging could be viewed simultaneously in the Radiology Information System (RIS) and Hospital Information System (HIS) system. Regarding the determination of lymph nodes, increased local uptake of pelvic and retroperitoneal lymph nodes was thought to be metastasis, while in MRI, a threshold of 1.0 cm short-axis node diameter for oval nodes and 0.8 cm for round nodes was used as a criterion for lymph node metastasis [19]. GTV includes definite primary prostate foci based on the abovementioned imaging basis; if accompanied by pelvic lymph node metastases, GTVn is outlined. CTV includes the prostatic + seminal vesicle bed (proximal 1–2.5 cm of seminal vesicle) and pelvic lymph nodes area. The pelvic lymphatic drainage area was outlined according to the NRG Oncology consensus contour atlas [20]. The above target areas were outlined manually on MRI and PET/MR fusion images based on axial T2-weighted MRI. The method of image acquisition and the sequence selected for target delineation is similar to a previous research project done by our group, which was based on assessing the clinical value of 18F-PSMA-1007 and 68 Ga-PSMA-11 PET/MRI in the gross tumour volume (GTV) delineation of radiotherapy for prostate cancer [21].

### Patient information

The individuals in this study had all been diagnosed with high-risk PCa. Basic and clinical information was collected for each patient, including age, TNM staging, Gleason score, preoperative PSA and pathology of surgical specimens. We considered the pathologic TNM (pTNM) classification or, when absent, the clinical TNM (cTNM) classification. The pathological results of all high-risk prostate pelvic lymph nodes obtained in our organization include both the number of positive lymph nodes and the total number of lymph nodes extracted. The surgical specimens of the pelvic lymph nodes are divided into seven groups to be submitted as packets. Including, common iliac nodes, left-internal iliac nodes, right-internal iliac nodes, left-external iliac nodes, right-external iliac nodes, obturator nodes, and presacral nodes. We divided lymph nodes into five groups for evaluation, including common iliac nodes, internal iliac nodes, external iliac nodes, obturator nodes, and presacral nodes.

### Statistical analysis

The data of CTV and GTVn volumes based on MRI and PET/MRI outlined by three observers and the conformity index (CI), the lesion coverage factor (LCF), and the Dice similarity coefficient (DSC) are presented as the mean ± standard deviation. Three methods of volumetric analysis, CI, LCF and DSC, were used to compare the correspondence between PET/MRI-CTV with MRI-CTV and PET/MRI-GTVn with MRI-GTVn. CI, used to determine the relative concordance between the two different modalities, was defined as A/B, where A and B represent two volumes delineated on MRI and PET/MRI, respectively (the same below). LCF, used to determine the percentage of overlap between the two volumes, was defined as (A ∩ B)/B, where A ∩ B represents the overlap between the two volumes (the same below). DSC, used to determine the similarities between the two datasets regarding both volumetric and spatial agreement, was defined as 2 × (A ∩ B)/(A + B), where (A + B) represents the sum of the absolute value of their volumes. The closer the CI result is to 1, the more similar the two volumes are. The closer the LCF and DSC result are to 1, the higher the degree of overlap between the two volumes. The Bland–Altman analysis between volumes delineated on PET/MRI and MRI was conducted.

The sensitivity and specificity of both MRI and PET/MRI imaging methods were evaluated for PLN, which were determined using pathological results as reference. The confusion matrix was presented. The paired sample Wilcoxon signed-rank tests were performed to compare the differences between the two groups of CTV and GTVn delineated by different methods (PET/MRI and MRI). The correlation between the two groups of CTV and GTVn was presented by scatter plot. Correlations were assessed using Pearson analysis. The McNemar test was used to compare the sensitivity and specificity of MRI and PET/MRI in detecting positive lymph nodes. 95% confidence intervals (95% CI) for sensitivity and specificity were calculated using Wilson score method [22]. SPSS 24.0 (IBM Corp, Armonk, NY, USA) was used for statistical analysis, and the ggplot, rms and foreign packages in R 3.4.3 (https://www.r-project.org/) were also used for statistical analysis. All statistical tests were 2-tailed, and P < 0.05 was considered statistically significant.

## Results

### Patient information

A total of 49 patients with high-risk prostate cancer underwent MRI and PSMA PET/MRI detection at Eastern Hospital between May 2020 and December 2021. The demographics of the patients are shown in Table 1. Patients had a median age of 72 years, of whom 14%, 70%, and 16% were at T2, T3, and T4 stages, respectively, and 27% of patients were at N1 stage. The mean value of PSA was approximately 16.81 ng/ml, and the majority of patients (47%) had a Gleason score of eight. All patients had adenocarcinoma confirmed by pathological biopsy. Thirty-seven patients had pathological examination of PLN.

**Table 1:** Patient demographics

Characteristic	Value
N	49
Age(y)	
Median (range)	72(42–89)
T Stage	
T2	7(14%)
T3	34(70%)
T4	8(16%)
N Stage	
N0	36(73%)
N1	13(27%)
M Stage	
M0	40(82%)
M1	9(18%)
PSA (ng/ml)	
Mean (std dev)	16.81 (19.11)
Gleason Score	
6	2(4%)
7	9(18%)
8	23(47%)
9	11(23%)
10	4(8%)

### Volume measurements

**Fig. 2:**
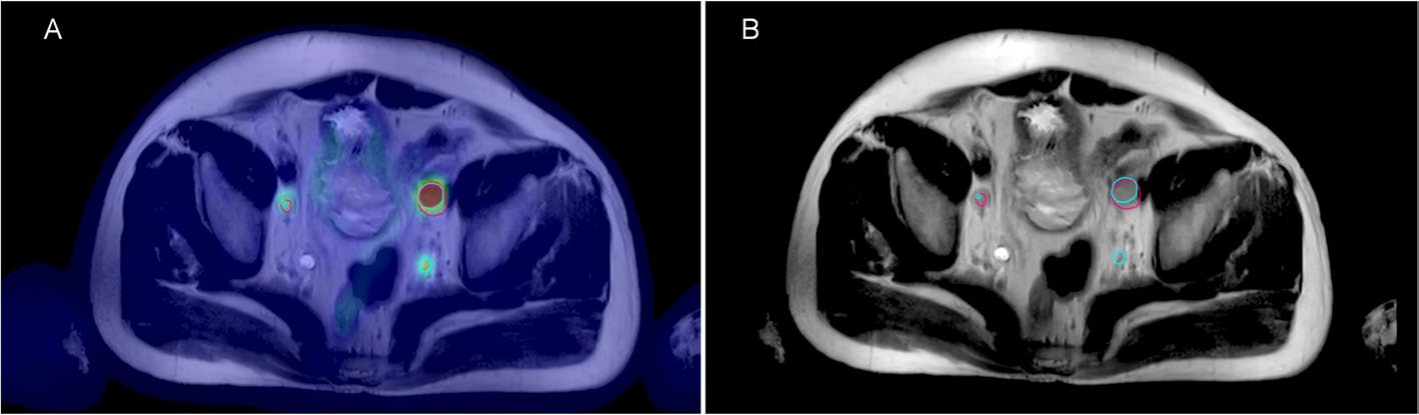
Target volume delineation for a 68-year-old man with high-risk PCa who was assumed three lymph node metastases based on PSMA Ligand PET/MRI (T2-weighted) leading to three GTVs (green line)(**A**). Target volume delineation based on MRI (T2-weighted) assuming two lymph node metastases leading to two GTVs (pink line) (**B**)

**Fig. 3:**
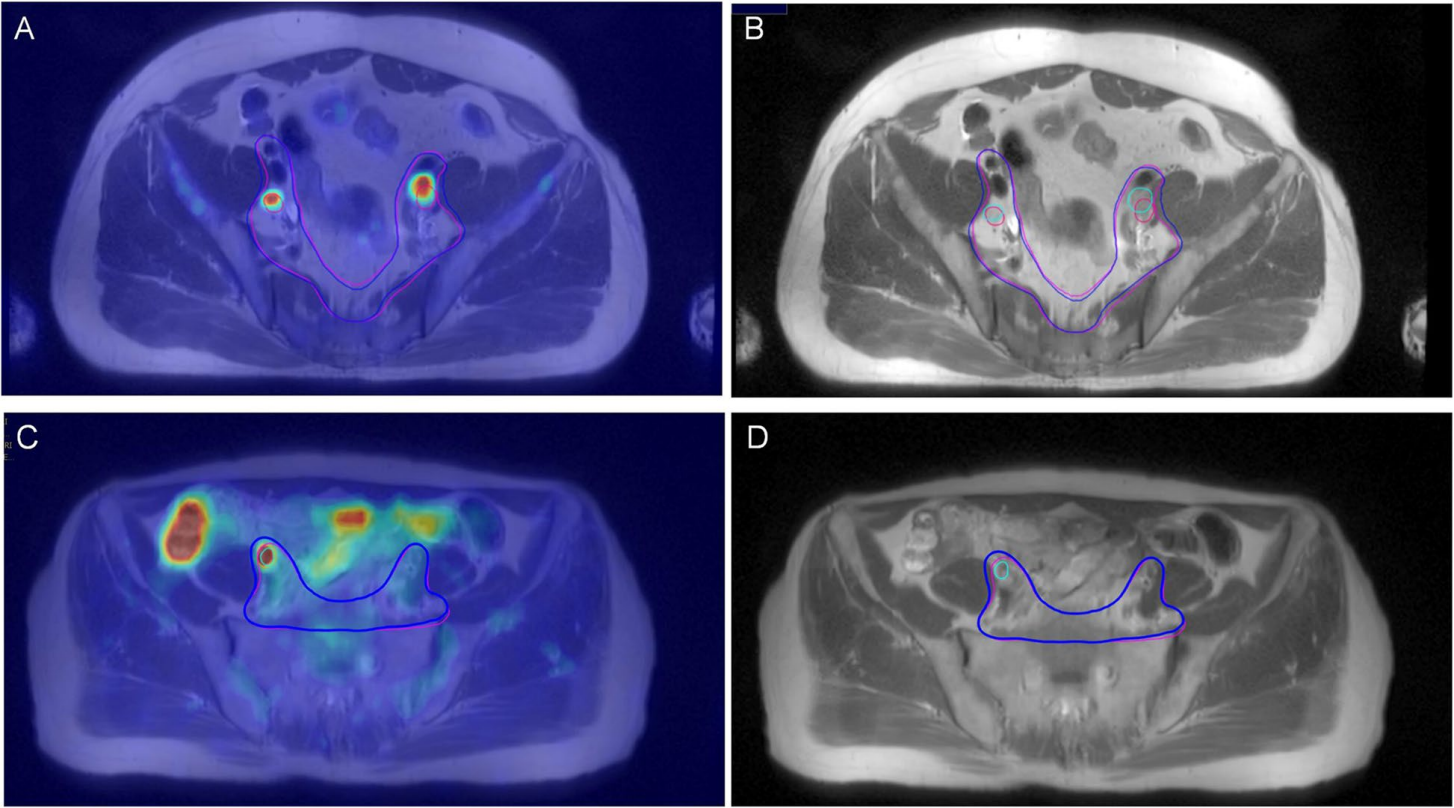
Target volume delineation for a 68-year-old man with high-risk PCa who was assumed two lymph node metastases based on PET/MRI (T2-weighted), leading to two GTVs (green line), resulting CTV in blue line (**A**). Target volume delineation based on MRI (T2-weighted) assuming two lymph node metastases leading to two GTVs (pink line), resulting CTV in pink line (**B**). Target volume delineation for a 67-year-old man with high-risk PCa who was assumed one lymph node metastasis based on PET/MRI (T2-weighted), leading to one GTV (green line), resulting CTV in blue line (**C**). Target volume delineation based on MRI (T2-weighted) assuming no lymph node metastasis, resulting CTV in pink line (**D**)

**Fig. 4:**
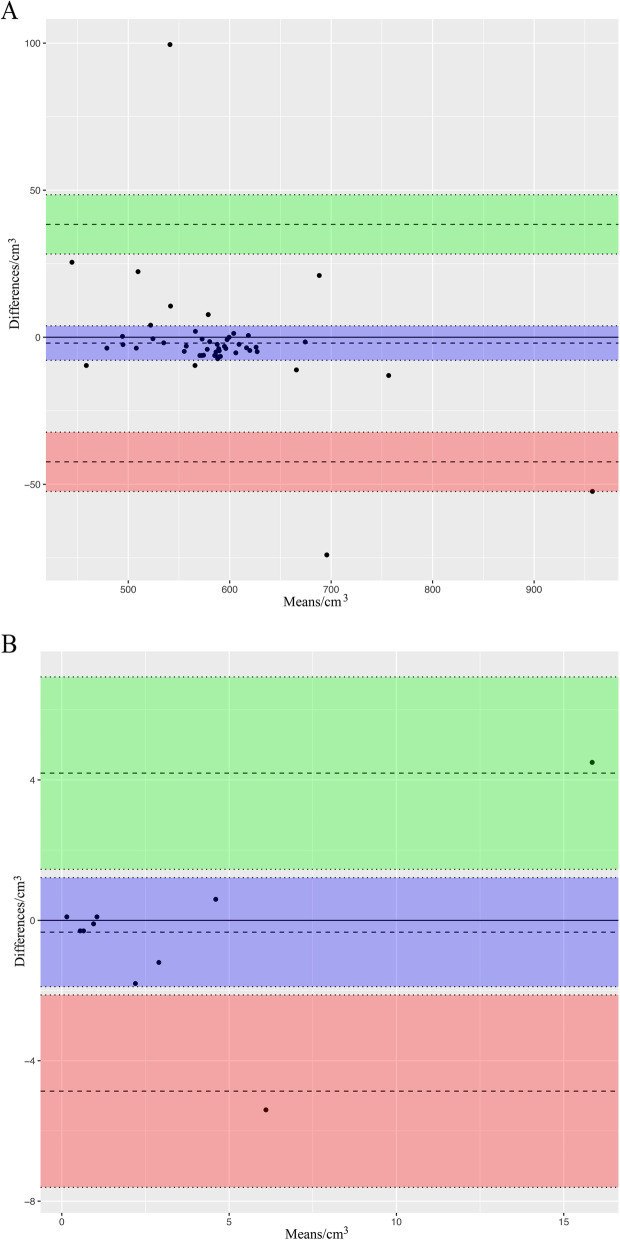
Bland–Altman analysis between volumes delineated on the two modalities for CTV(**A**) and for GTVn (**B**)

Figures 2 and 3 shows examples of delineation of CTV and GTVn of whole-pelvic radiotherapy measured under PSMA PET/MRI and MRI conditions. In our study, when each observer used MRI or PET/MRI to delineate the patient's target area, there would be a group of observation data and the recorded relevant parameters, including CI, LCF, and DSC. The mean values of CTV, GTVn, CI, DSC, and LCF based on MRI and PET/MRI measurements by three observers are shown in Supplement Tables 1 and 2. The Bland–Altman analysis between MRI and PET/MRI values for CTV and GTVn indicated mean differences of -1.98 and -0.34, respectively. The 95% CI for the difference was from -42.34 to 38.37, and from -4.87 to 4.19, respectively, as shown in Fig. 4. For CTV, the ordinates of 93.9% of the measured values were within the 95% CI, and for GTVn, the ordinates of 83.3% of the measured data were within the 95% CI, indicating that the data of the two groups had a good level of consistency. In addition, for CTV, the Bland–Altman analysis demonstrated that the data consistency was constant and would not change with the x-axis value (volume value). For GTVn, when the measured value of the x-axis was large, the data presented a large discrete type with a poor level of consistency.

**Table 2:** Clinical target volume measurement and parameter statistics

Characteristic (*n* = 49)	MRI-CTV (cc)	PET/MRI-CTV (cc)	Overlap volume (cc)	CI	LCF	DSC
Mean	579.3	580.3	552.8	0.999	0.953	0.954
SD	56.4	62.7	55.85	0.036	0.024	0.021

*CI* Conformity index, *LCF* Lesion-coverage factor, *DSC* Dice similarity coefficient, *CTV* Clinical target volume

The descriptive statistics of the target area, including the metrics of CTV, CI, DSC, and LCF, for the 49 patients were analysed (Table 2). The mean CTV for MRI-based contouring was 579.3 cm^3^, and the value of PET/MRI was 580.3 cm^3^. The CI was 0.999 ± 0.036, the LCF was 0.953 ± 0.024, and the DSC was 0.954 ± 0.021. Regarding volume and CI values, CTV-PET/MRI is comparable to CTV-MRI. The values of LCF and DSC were 0.953 and 0.954, respectively, indicating a high spatial overlap of the CTV based on the two imaging methods.

For the target area of positive lymph nodes in whole-pelvis radiation (GTVn), we observed pelvic lymph node metastasis in 12 of 49 cases based on MRI and PET/MRI, as described in Table 3 and Supplement Table 2. The average value of GTVn based on MRI and PET/MRI was 2.775 cm^3^ and 3.167 cm^3^, respectively. The mean CI, LCF, and DSC were 0.927 ± 0.621, 0.284 ± 0.272, and 0.321 ± 0.235, respectively. Regarding volume size, the mean value of MRI-based GTVn was slightly smaller than that based on PET/MRI, with a CI of 0.927. The heterogeneity of MRI- and PET/MRI-based lymph node profiles was high according to the value of LCF and DSC.

**Table 3:** Nodal gross tumor volume measurement and parameter statistics

Characteristic (*n* = 12)	MRI-GTVn(cc)	PET/MRI-GTVn(cc)	Overlap volume (cc)	CI	LCF	DSC
Mean	2.775	3.167	1.433	0.927	0.284	0.295
SD	5.047	4.118	2.854	0.621	0.272	0.242

*CI* Conformity index, *LCF* Lesion-coverage factor, *DSC* Dice similarity coefficient, *GTVn* Nodal gross tumor volume

**Fig. 5:**
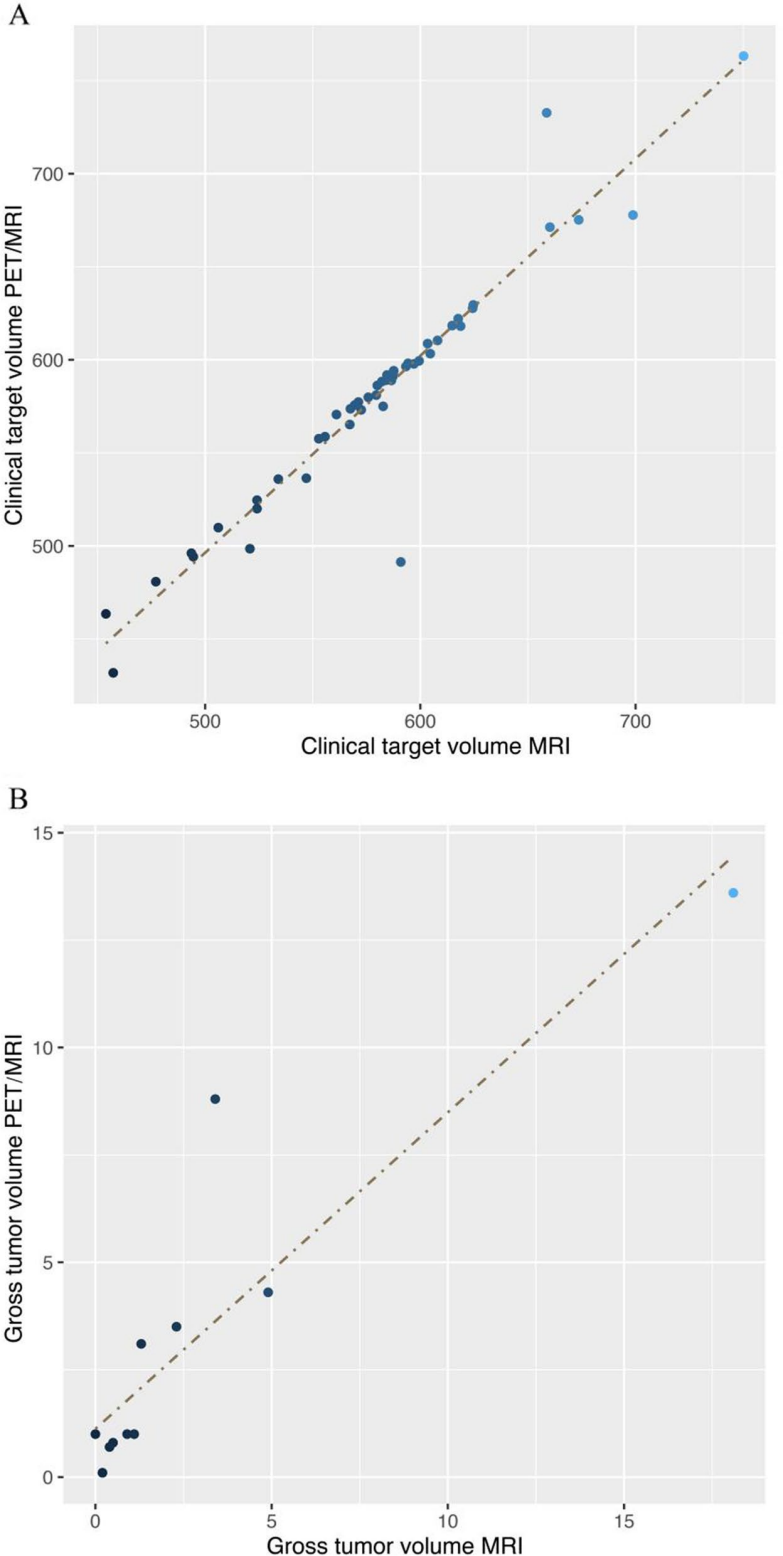
PSMA PET/MRI-CTV versus MRI-CTV (**A**); PSMA PET/MRI-GTVn versus MRI-GTVn (**B**)

The results of the paired sample Wilcoxon signed-rank tests showed non-significant differences in the CTV and GTVn based on MRI and PET/MRI images (*P* = 0.660, *P* = 0.650). According to the results of the correlation analysis, there was a statistically significant link between the MRI-CTV volume and PET/MRI-CTV volume (*P* = 0.005), as well as between MRI-GTVn volume and PET/MRI-GTVn volume (*P* < 0.001) (Fig. 5).

### Diagnostic sensitivity and specificity for the detection of pelvic lymph node

**Fig. 6:**
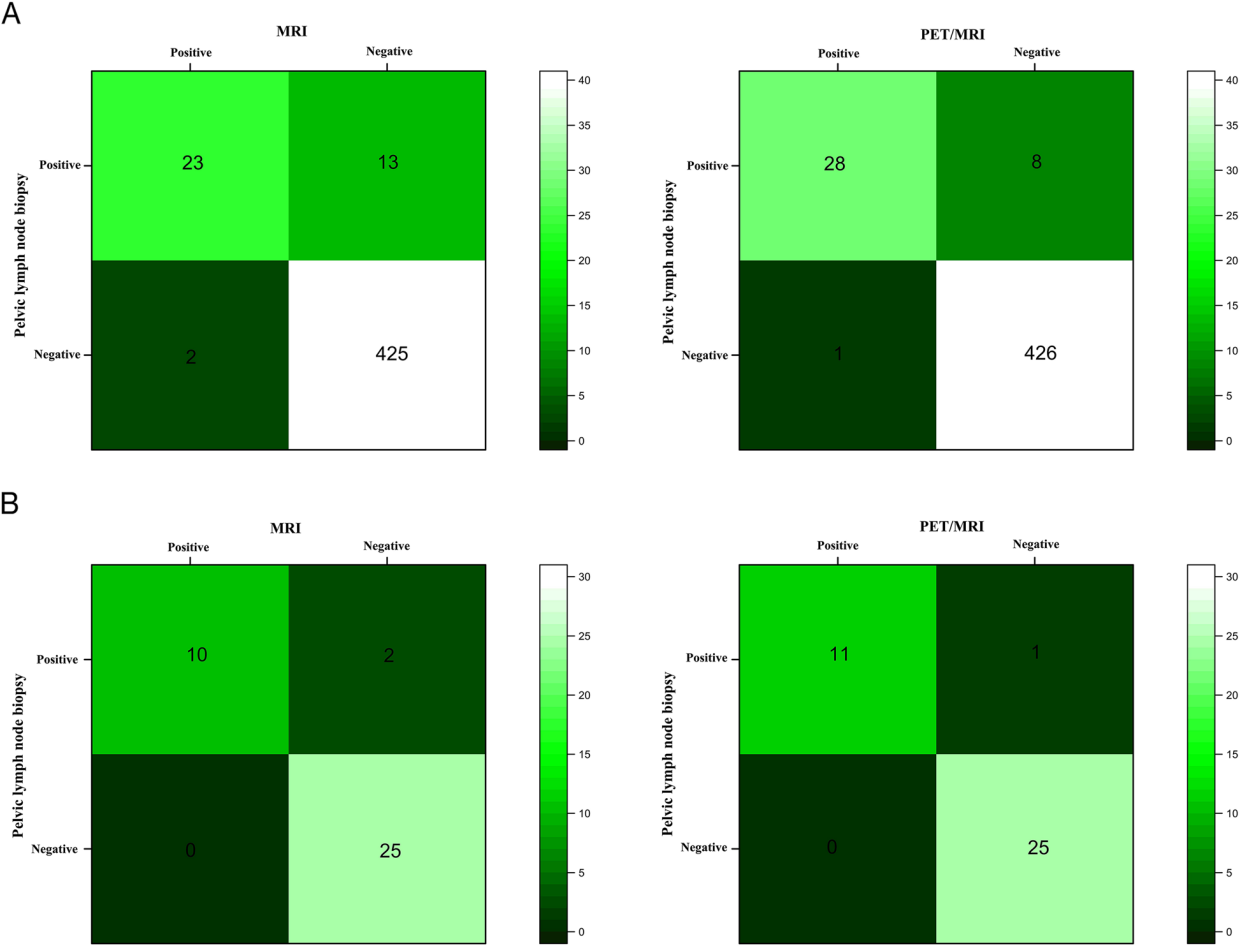
Confusion matrix results of MRI and PET/MRI for lymph node-based analysis (**A**) and for patient-based analysis (**B**)

Of the 49 patients included in the study, 463 lymph nodes from 37 patients had pathological examination of PLN, and the demographic and clinical characteristics of the above patients are described in Supplement Table 3. All of these patients underwent radical prostatectomy (RP) and pelvic lymph node dissection (PLND). Extended pelvic lymph node dissection (EPLND) was chosen in 27 of these patients. In Fig. 6, we present a confusion matrix of the final detection results. In the lymph node-based analysis, a total of 28/36 (77.8%) positive lymph nodes were detected by PET/MRI, while 8 lymph nodes were classified as false negatives (22.2% of abnormal lymph nodes were missed). A total of 23/36 (63.9%) positive lymph nodes were correctly detected by MRI, while 13 lymph nodes were classified as false negatives (36.1% of abnormal lymph nodes were missed). In the patient-based analysis, among 37 patients, neither MRI nor PET/MRI detected positive lymph nodes in 25 patients without pelvic lymph node metastasis (PLNM), and for 12 patients with definite pelvic lymph node pathology biopsies, PET/MRI identified 11/12 (91.7%) patients with PLNM, while MRI identified 10/12 (83.3%) patients with PLNM. The sensitivity and specificity of PET/MRI vs. MRI for lymph node detection at two levels of analysis are shown in Table 4. A comparison of the efficacy of PET/MRI and MRI for PLNM revealed a significant difference between the two methods (*P* = 0.011). In Supplement Table 4, the relationship between imaging diagnosis and pathology of pelvic lymph nodes (divided into five groups, including common iliac nodes, internal iliac nodes, external iliac nodes, obturator nodes, and presacral nodes) is reflected.

**Table 4:** PSMA PET/MRI has higher sensitivity and specificity for detecting pelvic lymph node metastasis when compared to MRI. (A: lymph node-based analysis; B: patient-based analysis)

Parameter		Sensitivity % (95% CI)	Specificity % (95% CI)
A	PET/MRI	77.78 (61.91 to 88.28)	99.76 (98.68 to 99.96)
	MRI	63.89 (75.03 to 97.78)	99.53 (98.31 to 99.87)
B	PET/MRI	91.67 (64.61 to 98.51)	100.00 (86.68 to 100.00)
	MRI	83.33 (55.20 to 95.30)	100.00 (86.68 to 100.00)

The 95% CI for the sensitivity and specificity estimates were calculated using Wilson's score method

## Discussion

For high-risk prostate cancer patients, this is the first study to examine PSMA PET/MRI-based target volumes (TVs) for whole-pelvis radiation. This is also the first study comparing PSMA PET/MRI and MRI-based radiotherapy target volumes. We found that the GTVn based on PSMA PET/MRI differs from that based on MRI, with existing spatial heterogeneity between them. PSMA PET/MRI can identify more lymph node metastasis or even change the tumour stage of some patients, and it also reduces the probability of false-positive lymph nodes and changes the TVs in radiotherapy planning (RTP) to protect normal tissues. The difference between CTVs based on PSMA PET/MRI and MRI was not significant, indicating that the ability to identify the tissue structure of the pelvic drainage area was similar for PSMA PET/MRI and MRI.

More than 30–40% of high-risk PCa patients present with PLNM during pelvic lymph node dissection and staging [23]. The treatment of PLN with external beam radiation therapy (RT) is a frequent component of the management of patients with prostate cancer. Whole pelvic radiation therapy (WPRT) is a common practice for men receiving prostate radiotherapy for high-risk disease, clinical lymph node-positive disease, and postprostatectomy [5, 24, 25].The consensus atlas for pelvic nodal contouring in the clinical target area for WPRT was newly revised in 2021. In our research, CTV was contoured based on MRI and PET/MRI according to the above principles. No statistically significant differences were found when comparing the results, no matter the volume size or spatial coincidence. Based on the Bland–Altman analysis, the MRI-CTV and PET/MRI-CTV had good consistency without changing with the size of the CTV. This shows that the CTV delineated based on the two methods of whole pelvic radiotherapy for prostate cancer is comparable. Although the boundary of CTV may differ due to the difference in GTVn (as depicted in Fig. 3), the range of CTV was much larger than GTVn, so that the difference caused by GTVn cannot be clearly reflected. In a word, the results reflect the high similarity of the CTV obtained by the two methods. Ingrid White et al. noted that MRI provides superior soft tissue contrast compared to CT in RTP for rectal cancer, resulting in a clearer delineation of the boundaries of the target areas and reducing the volume due to observer uncertainty, as demonstrated by the reduced margins of both CTV and PTV in the corresponding adapted radiotherapy [26]. Based on our research results, the CTVs of PET/MRI and MRI are similar. Therefore, despite the lack of research comparing the difference between targets delineated based on CT and PET/MRI, we believe that PET/MRI has advantages similar to those of MRI in target delineation for radiotherapy, namely, based on the excellent soft tissue resolution it provides, doctors could delineate the boundary of the target areas more clearly.

PSMA-targeted PET provides better detection of metastases than conventional imaging (i.e., CT, multiparametric MRI, and 18F-based PET-CT [flucilloflox and choline]). Several studies have confirmed that 68 Ga-PSMA PET-CT has higher sensitivity and specificity in identifying pelvic nodes and/or distant metastases with biopsy-proven high-risk PCa than CT or bone scans. Sawicki et al. found that 68 Ga-PSMA PET-CT detected lesions missed by WB-MRI in patients with biochemical failure after radical prostate cancer surgery [27–31]. Another study of a high-risk prostate cancer group showed that PSMA PET-CT resulted in improved overall staging in 23.9% of patients with negative conventional imaging (CT or MRI) [32]. Even in the diagnosis of small lymph nodes, PSMA PET also had a specificity of 95% for diagnosis [33, 34]. An article evaluating the use of [68 Ga] Ga-PSMA-11 PET (PSMA-PET)/MRI in the staging of primary tumour-node-metastasis in prostate cancer affirmed its excellent accuracy (93% accuracy at N1 stage) and found that it could change the treatment strategy in 28.7% of patients [16]. In this paper, compared to MRI, PSMA PET/MR had high sensitivity and specificity, with significant differences between the two methods (*P* = 0.011). Of the 49 high-risk prostate cancer patients, 21 patients (42.9%) had N1/M1 disease, of whom 1 patient was previously N0 and 7 patients had bone metastases on PSMA PET/MRI. Similar results were confirmed in other articles. The high sensitivity and specificity of PSMA PET/MRI changes the clinical stage of patients, which may affect the treatment plan of patients [35].

Integration of PET/CT in radiotherapy planning is common in many cancer types [10], and PET/MRI is now increasingly being used in RTP [36–41]. In prostate cancer, several studies have also evaluated the use of PSMA PET/MRI in the radiotherapy of primary and recurrent prostate cancer [16, 42, 43]. We are the first to investigate the application of PSMA PET/MRI in the GTVn of radiotherapy, and we discovered that when compared to MRI, PSMA PET/MRI has altered the RTP. The spatial analysis index indicated that the overlap between the two GTVn was low, and when combined with the analysis of sensitivity and specificity for lymph nodes, we confidently concluded that the GTVn contouring on PSMA PET/MRI is more accurate and may help to reduce lymph node recurrence. In combination with a recent research effort conducted by our group, which showed that it was feasible to visually delineate GTV on PSMA PET/MRI in PCa radiotherapy [21]. Our investigation has shown that it is feasible to delineate GTVn and CTV on PSMA PET/MRI. Therefore, we have emphasized the utility of PET/MRI fusion images in delineating prostate cancer radiotherapy.

It is necessary to acknowledge some limitations. The nature of retrospective studies may have introduced unnoticed statistical bias in adherence. CT is the most commonly used imaging technique in radiotherapy treatment planning. This paper only compares PET/MRI and MRI, leaving CT out of the equation. It is basically impossible to obtain the results of node-to-node rad path correlation, so we grouped pelvic lymph nodes into five groups for a matching analysis between pathology and imaging, which may bias the results of the diagnosing efficacy of imaging methods (PET/MRI or MRI). In addition, we only analysed a small sample size, specifically, the CTV of 49 patients and the GTVn of 12 patients. As a result, the practical application value of PSMA PET/MRI requires further investigation in large, well-designed, randomized, controlled trials.

## Conclusion

PSMA PET/MRI-GTVn is more accurate in whole pelvic radiotherapy for high-risk prostate cancer patients because PSMA PET/MRI has higher sensitivity, specificity and diagnostic accuracy than MRI in identifying PLNM. The CTV of whole pelvic radiotherapy determined by PSMA PET/MRI and MRI is comparable. Eight high-risk prostate cancer patients who finished PSMA PET/MRI changed their N or M stage. The use of PSMA PER/MRI aids in the realization of more individualized and precise radiotherapy.

## Supplementary Information

**Additional file 1.** 

## Data Availability

The datasets used and/or analysed during the current study are available from the corresponding author on reasonable request.

## Abbreviations

PCa: Prostate cancer
RTP: Radiotherapy planning
PSMA: Prostate-specific membrane antigen
CTV: Clinical target volume
GTVn: Nodal gross tumour volume
WPRT: Whole pelvic radiation therapy
PLNM: Pelvic lymph node metastasis
RP: Radical prostatectomy
PLN: Pelvic lymph node
PLND: Pelvic lymph node dissection
EPLND: Extended pelvic lymph node dissection
CI: Conformity index
LCF: Lesion coverage factor
DSC: Dice similarity coefficient
EAU: European Association of Urology
