# Supplementary material for: Variability of radiotherapy volume delineation: PSMA PET/MRI and MRI based clinical target volume and lymph node target volume for high-risk prostate cancer
Source: Cancer Imaging. 2023 Jan 4;23:1. doi: 10.1186/s40644-022-00518-7 (PMC9811734; doi:10.1186/s40644-022-00518-7)
Supplement: Supplementary file 1 — Additional file 1. [file 40644_2022_518_MOESM1_ESM.docx]

**Supplement Table 1** Average patient volume measurement (CTV) and related parameter values as determined by three observers

| Patient Number  (n=49) | MRI-CTV (cc) | PET/MRI-CTV (cc) | Overlap volume (cc) | CI | LCF | DSC |
| --- | --- | --- | --- | --- | --- | --- |
| 1 | 477.1 | 480.8 | 448.6 | 0.992 | 0.933 | 0.937 |
| 2 | 660.2 | 671.3 | 635.8 | 0.983 | 0.947 | 0.955 |
| 3 | 569.5 | 575.7 | 552.3 | 0.989 | 0.959 | 0.965 |
| 4 | 524.1 | 524.6 | 495.7 | 0.999 | 0.945 | 0.945 |
| 5 | 624.3 | 627.7 | 575.1 | 0.995 | 0.916 | 0.919 |
| 6 | 593.3 | 596.4 | 571.1 | 0.995 | 0.958 | 0.96 |
| 7 | 571.2 | 577.3 | 540.2 | 0.989 | 0.936 | 0.941 |
| 8 | 750.2 | 763.2 | 692.6 | 0.983 | 0.907 | 0.915 |
| 9 | 673.6 | 675.2 | 656.5 | 0.998 | 0.972 | 0.973 |
| 10 | 624.6 | 629.5 | 607.6 | 0.992 | 0.965 | 0.969 |
| 11 | 586.5 | 588.9 | 579.9 | 0.996 | 0.985 | 0.987 |
| 12 | 584.5 | 591.8 | 548.0 | 0.988 | 0.926 | 0.932 |
| 13 | 584.0 | 589.0 | 559.2 | 0.992 | 0.949 | 0.953 |
| 14 | 579.5 | 581.0 | 559.9 | 0.997 | 0.964 | 0.965 |
| 15 | 572.5 | 573.1 | 530.9 | 0.999 | 0.926 | 0.927 |
| 16 | 608.0 | 610.4 | 587.4 | 0.996 | 0.962 | 0.964 |
| 17 | 552.8 | 557.6 | 532.9 | 0.991 | 0.956 | 0.96 |
| 18 | 617.6 | 622.1 | 592.1 | 0.993 | 0.952 | 0.955 |
| 19 | 555.7 | 558.7 | 536.6 | 0.995 | 0.96 | 0.963 |
| 20 | 587.2 | 591.1 | 580.9 | 0.993 | 0.983 | 0.986 |
| 21 | 603.4 | 608.7 | 557.7 | 0.991 | 0.916 | 0.92 |
| 22 | 614.8 | 618.4 | 601.2 | 0.994 | 0.972 | 0.975 |
| 23 | 604.6 | 603.3 | 573.6 | 1.002 | 0.951 | 0.95 |
| 24 | 506.1 | 509.8 | 472.3 | 0.993 | 0.926 | 0.93 |
| 25 | 547.0 | 536.4 | 516.1 | 1.020 | 0.962 | 0.953 |
| 26 | 590.9 | 491.4 | 487.9 | 1.202 | 0.993 | 0.902 |
| 27 | 567.5 | 573.7 | 560.6 | 0.989 | 0.977 | 0.982 |
| 28 | 698.8 | 677.8 | 656.9 | 1.031 | 0.969 | 0.954 |
| 29 | 584.7 | 591.3 | 538.6 | 0.989 | 0.911 | 0.916 |
| 30 | 618.7 | 618.1 | 586.5 | 1.001 | 0.949 | 0.948 |
| 31 | 567.2 | 565.2 | 560.1 | 1.004 | 0.991 | 0.989 |
| 32 | 597.0 | 597.8 | 579.5 | 0.999 | 0.969 | 0.97 |
| 33 | 587.4 | 592.0 | 552.3 | 0.992 | 0.933 | 0.937 |
| 34 | 582.7 | 575.0 | 542.2 | 1.013 | 0.943 | 0.937 |
| 35 | 494.5 | 494.2 | 468.3 | 1.001 | 0.948 | 0.947 |
| 36 | 575.8 | 579.9 | 561.3 | 0.993 | 0.968 | 0.971 |
| 37 | 520.8 | 498.5 | 480.0 | 1.045 | 0.963 | 0.942 |
| 38 | 524.1 | 520.0 | 511.9 | 1.008 | 0.984 | 0.981 |
| 39 | 477.1 | 480.8 | 448.6 | 0.992 | 0.933 | 0.937 |
| 40 | 493.6 | 496.1 | 474.5 | 0.995 | 0.956 | 0.959 |
| 41 | 658.7 | 732.7 | 644.3 | 0.899 | 0.879 | 0.926 |
| 42 | 587.6 | 594.1 | 582.4 | 0.989 | 0.98 | 0.986 |
| 43 | 599.4 | 599.4 | 572.2 | 1.000 | 0.955 | 0.955 |
| 44 | 582.1 | 588.3 | 559.0 | 0.989 | 0.95 | 0.955 |
| 45 | 453.9 | 463.5 | 449.8 | 0.979 | 0.97 | 0.981 |
| 46 | 594.3 | 598.1 | 576.3 | 0.994 | 0.964 | 0.967 |
| 47 | 561.0 | 570.6 | 548.5 | 0.983 | 0.961 | 0.969 |
| 48 | 534.0 | 535.9 | 495.2 | 0.996 | 0.924 | 0.926 |
| 49 | 457.3 | 431.8 | 425.2 | 1.059 | 0.985 | 0.956 |
| CI, conformity index; LCF, lesion-coverage factor; DSC, dice similarity coefficient; CTV, clinical target volume. | | | | | | |

**Supplement Table 2** Average patient volume measurement (GTVn) and related parameter values as determined by three observers

| Patient Number (n=12) | MRI-GTVn(cc) | PET/MRI-GTVn(cc) | Overlap volume (cc) | CI | LCF | DSC |
| --- | --- | --- | --- | --- | --- | --- |
| 1 | 0.2 | 0.1 | 0.0 | 2.000 | 0.000 | 0.000 |
| 2 | 0.4 | 0.7 | 0.1 | 0.571 | 0.143 | 0.182 |
| 3 | 18.1 | 13.6 | 10.1 | 1.331 | 0.743 | 0.637 |
| 4 | 0.0 | 1.0 | 0.0 | 0.000 | 0.000 | 0.000 |
| 5 | 4.9 | 4.3 | 2.7 | 1.140 | 0.628 | 0.587 |
| 6 | 0.9 | 1.0 | 0.4 | 0.900 | 0.400 | 0.421 |
| 7 | 0.5 | 0.8 | 0.2 | 0.625 | 0.250 | 0.308 |
| 8 | 1.3 | 3.1 | 0.4 | 0.419 | 0.129 | 0.182 |
| 9 | 3.4 | 8.8 | 1.9 | 0.386 | 0.216 | 0.311 |
| 10 | 2.3 | 3.5 | 0.7 | 0.657 | 0.200 | 0.241 |
| 11 | 0.2 | 0.1 | 0.0 | 2.000 | 0.000 | 0.000 |
| 12 | 1.1 | 1.0 | 0.7 | 1.100 | 0.700 | 0.667 |
| CI, conformity index; LCF, lesion-coverage factor; DSC, dice similarity coefficient; GTVn, nodal gross tumor volume. | | | | | | |

**Supplement Table 3** Demographic information of high-risk prostate cancer patients with pelvic lymph node biopsy results

| Characteristic | Value |
| --- | --- |
| N | 37 |
| Age(y) |  |
| Median (range) | 72(67-89) |
| T Stage |  |
| T2 | 4(10.8%) |
| T3 | 28(75.7%) |
| T4 | 5(13.5%) |
| N Stage |  |
| N0 | 23(62.2%) |
| N1 | 14(37.8%) |
| M Stage |  |
| M0 | 26(70.3%) |
| M1 | 11(29.7%) |
| PSA (ng/ml) |  |
| Mean (std dev) | 16.7 (21.37) |
| Gleason Score |  |
| 6 | 1(3.0%) |
| 7 | 6(16.0%) |
| 8 | 18(49.0%) |
| 9 | 10(27.0%) |
| 10 | 2(5.0%) |
| Number of positive lymph nodes |  |
| 0 | 27(73.0%) |
| 1 | 5(13.5%) |
| ≥2 | 5(13.5%) |

**Supplement Table 4** Corresponding the pathology of the distribution of lymph nodes to imaging diagnoses (PET/MRI or MRI)

| IMAGING | LYMPH NODES (number of cases) | | | | |
| --- | --- | --- | --- | --- | --- |
|  | COMMON ILIAC | INTERNAL ILIAC | OBTURATOR | EXTERNAL ILIAC | PRESACRAL |
| MRI- bio+ | 3 | 3 | 2 | 5 | 0 |
| MRI+ bio- | 1 | 0 | 0 | 0 | 1 |
| MRI+ bio+ | 3 | 15 | 2 | 2 | 1 |
| PET/MRI- bio+ | 1 | 1 | 2 | 4 | 0 |
| PET/MRI+ bio- | 0 | 0 | 1 | 0 | 0 |
| PET/MRI+ bio+ | 5 | 17 | 2 | 3 | 1 |
